# Isolation of a Ceftazidime-Avibactam-Resistant *bla*_KPC-71_-Positive Klebsiella pneumoniae Clinical Isolate

**DOI:** 10.1128/spectrum.01840-21

**Published:** 2022-01-26

**Authors:** Siquan Shen, Qingyu Shi, Renru Han, Yan Guo, Yang Yang, Shi Wu, Dandan Yin, Fupin Hu

**Affiliations:** a Institute of Antibiotics, Huashan Hospital, Fudan University, Shanghai, China; b Key Laboratory of Clinical Pharmacology of Antibiotics, Ministry of Health, Shanghai, China; National University Hospital

**Keywords:** KPC-2, KPC-71, ST11, ceftazidime/avibactam

## LETTER

Carbapenem-resistant Klebsiella pneumoniae with the characteristics of extensive drug resistance has been increasingly reported worldwide, accompanied by high mortality rates because the available therapeutic regimens for treatment of infections are limited (1). According to data from the China Antimicrobial Surveillance Network, the rates of K. pneumoniae resistance to meropenem and imipenem constantly increased from 2.9% and 3.0%, respectively, in 2005 to 27.1% and 25.5% in 2021 (2). In Europe, carbapenem-resistant K. pneumoniae strains are most widespread in the Mediterranean and Balkan countries, with prevalence rates of 60% in Greece and 40% in Italy (3).

Production of Klebsiella pneumoniae carbapenemase (KPC) is the most common mechanism of K. pneumoniae resistance to carbapenems (4), involving almost entirely *bla*_KPC-2_ in China (4). Currently, ceftazidime-avibactam is one of the best available therapeutic options for infections caused by such isolates. Several studies reported that ceftazidime-avibactam presents excellent *in vitro* activity for the effective inhibition of serine-carbapenemase (KPC and OXA-48-like), and it was then endorsed as a first-line drug for the treatment of carbapenem-resistant *Enterobacteriaceae* (CRE)-related infections in China in 2019. However, following the wide clinical application of ceftazidime-avibactam, K. pneumoniae acquired resistance rapidly. Currently, 98 *bla*_KPC_ subtypes have been reported in the world (5); all of the novel *bla*_KPC_ variants reported in China were mutated from *bla*_KPC-2_, while those in the United States were mainly from *bla*_KPC-3_ (6–9).

In this study, we describe the characterization of a ceftazidime-avibactam-resistant *bla*_KPC-71_-positive K. pneumoniae clinical isolate. Forty-five days after femoral neck fixation, a 71-year-old female patient had an intermittent fever for more than 1 month, and multidrug-resistant K. pneumoniae was isolated from a sputum sample. The isolate was resistant to expanded-spectrum cephalosporins (ceftazidime and cefepime), imipenem, meropenem, amikacin, ciprofloxacin, and trimethoprim-sulfamethoxazole but was susceptible to ceftazidime-avibactam and tigecycline. Later, the patient was admitted to Huashan Hospital, Fudan University (eastern China), and ceftazidime-avibactam (2.5 g every 8 h) was used for treatment of the infection after her hospitalization. Ceftazidime-avibactam-resistant K. pneumoniae HS645 was isolated from sputum after 3 days. The therapeutic regimen was switched to ceftazidime-avibactam (2.5 g every 8 h) plus aztreonam (1 g every 8 h) 3 days later, and K. pneumoniae strain HS826, carrying *bla*_KPC-2_, was isolated at the same time; tigecycline (first dose of 100 mg and then continuous infusion of 50 mg every 12 h) was used for the treatment of these two strains. The patient recovered and was discharged 21 days after admission. Identification of the isolate at the species level was carried out using a Vitek mass spectrometer. Antimicrobial susceptibility testing was carried out with the broth microdilution method, and results were interpreted according to the CLSI breakpoints (10). The results indicated that K. pneumoniae HS645 was resistant to expanded-spectrum cephalosporins (ceftazidime and cefepime), piperacillin-tazobactam, and ceftazidime-avibactam but retained susceptibility to imipenem and intermediate susceptibility to meropenem. The strain was also resistant to ciprofloxacin and trimethoprim-sulfamethoxazole while being susceptible to amikacin and tigecycline (Table 1). An electroporation experiment was performed to explore the characteristics of the KPC-71-carrying plasmid. Escherichia coli DH5α was used as the recipient strain. The transformant was positive for *bla*_KPC-71_, increasing the MICs of ceftazidime-avibactam, ceftazidime, and cefepime for the transformant by at least 32-fold, 128-fold, and 64-fold, respectively, compared with the recipient E. coli DH5α (Table 1).

**TABLE 1:** Susceptibility of K. pneumoniae clinical isolates, transformant, and recipient to antimicrobial agents

Strain	β-Lactamase gene(s)	Other resistance genes	MIC (mg/L)^*a*^									
			CZA	IPM	MEM	FEP	CAZ	TZP	AMK	SXT	CIP	TGC
K. pneumoniae HS826^*b*^	*bla*_KPC-2_		4	64	>64	>128	>32	>256	>128	>32	>8	2
K. pneumoniae HS645	*bla*_CTX-M-65_, *bla*_LAP-2_, *bla*_KPC-71_, *bla*_SHV-12_, *bla*_TEM-1B_	*qnrS1*, *aadA2b*, *rmtB*, *tet*(A), *fosA*, *sul2*	>128	0.5	2	>128	>32	>256	>128	>32	>8	2
E. coli DH5α-HS645-T	*bla*_CTX-M-65_, *bla*_KPC-71_, *bla*_SHV-12_, *bla*_TEM-1B_	*rmtB*	4	0.125	≤0.03	8	>32	8	>128	≤0.05	≤0.06	0.125
E. coli DH5α			≤0.125	≤0.06	≤0.03	≤0.125	≤0.25	≤4	≤8	≤0.05	≤0.06	≤0.06

a CZA, ceftazidime-avibactam; IPM, imipenem; MEM, meropenem; FEP, cefepime; CAZ, ceftazidime; TZP, piperacillin-tazobactam; AMK, amikacin; SXT, trimethoprim-sulfamethoxazole; CIP, ciprofloxacin; TGC, tigecycline.

b K. pneumoniae HS826 production of KPC-2 was confirmed by PCR-based sequencing.

To further investigate the mechanisms of resistance, the K. pneumoniae clinical isolate was subjected to whole-genome sequencing (WGS) using an Illumina MiSeq platform (Illumina Inc.) with a paired-end approach (2 × 300 bp). *De novo* assembly of WGS data was performed using SPAdes software (http://cab.spbu.ru/software/spades). WGS analysis revealed that K. pneumoniae HS645 belonged to sequence type 11 (ST11), and the following acquired resistance determinants were detected: resident *bla*_KPC-71_, *bla*_CTX-M-65_, *bla*_SHV-12_, *bla*_LAP-2_, and *bla*_TEM-1B_ and resistance determinants for aminoglycosides (*aadA2b* and *rmtB*), fluoroquinolone (*qnrS1*), tetracycline [*tet*(A)], fosfomycin (*fosA*), and sulfonamide (*sul2*) (Table 1). Plasmid DNA sequencing localized *bla*_KPC-71_ on a plasmid about 60 kb in length, which contains the resistance genes on drug resistance gene islands similar to that reported in Hunan Province in China (11). Compared with KPC-2, KPC-71 has a mutation of Ser182dup.

Novel KPC variants have emerged rapidly worldwide in recent years. The number of novel KPC variants reported since 2020 exceeds the sum of the previous 17 years (5). Problems have arisen in clinical microbiology because of these novel KPC variants, owing to their inconspicuous characteristic of carbapenem resistance and lack of an effective detection method. Routine detection methods for carbapenemase, including the Carba NP test, the modified carbapenem inactivation method (mCIM)/EDTA-modified CIM (eCIM) recommended by the CLSI, the 3-aminophenylboronic acid (APB)/EDTA synergy test, and the NG-Test Carba 5, usually show false-negative results for these isolates (6). These novel KPC variants may fail to grow on selective chromogenic medium (6). Interestingly, *bla*_KPC-71_ variants conferred resistance to ceftazidime-avibactam but restored susceptibility to imipenem.

Our study indicated that KPC-71 is a new variant of KPC-2 (Ser182dup) that confers a ceftazidime-avibactam-resistant phenotype associated with susceptibility or decreased resistance to carbapenems, compared with KPC-2. The mechanism of resistance to ceftazidime-avibactam for KPC-71 has not been kinetically investigated, but the mutation seems to increase the enzyme’s affinity for ceftazidime and decrease inhibition by avibactam (12, 13). To the best of our knowledge, this is the first report of a ceftazidime-avibactam-resistant *bla*_KPC-71_-producing K. pneumoniae clinical isolate in China. Our study further confirmed that novel KPC variants originally from KPC-2 can be selected following exposure to ceftazidime-avibactam, and it underscored that similar mutants have relevant diagnostic implications, since they might be missed by routine detection methods for carbapenemase, while might affect the prediction of ceftazidime-avibactam susceptibility, resulting in incorrect clinical usage.

KPC-71 harbored by a ceftazidime-avibactam-resistant K. pneumoniae strain is a microcosm of the evolution of KPC-2 mutations. To date, more and more novel KPC variants have been reported, such as KPC-33 and KPC-74 (7, 14). KPC-74, with a deletion of 6 nucleotides at positions 712 to 717, compared with *bla*_KPC–2_, showed resistance to ceftazidime-avibactam. Similarly, the results of the NG-Test Carba-5, a commercial rapid detection method for *bla*_KPC–2_, were negative for *bla*_KPC–74_ (14). Thus, a rapid and accurate detection method needs to be developed for the detection of *bla*_KPC_ variants. In terms of treatment, a novel therapeutic regimen is urgently needed as salvage therapy for infections caused by ceftazidime-avibactam-resistant K. pneumoniae strains, such as meropenem-vaborbactam (15), imipenem-relebactam, eravacycline, and cefiderocol. Clinical microbiology laboratories also should seek new diagnostic approaches to detect novel KPC variants. Furthermore, large-scale epidemiological investigations of novel KPC variants and surveillance of ceftazidime-avibactam resistance among *Enterobacterales* clinical isolates, especially for K. pneumoniae, should be carried out in a timely manner to combat the spread of resistance.

### Data availability.

The nucleotide sequence of the plasmid carrying *bla*_KPC-71_ is available in GenBank under accession number OK315339.
